# Uptake of orthodontic treatment by children and adolescents in Germany. Results of the cross-sectional KiGGS Wave 2 study and trends

**DOI:** 10.17886/RKI-GBE-2018-101

**Published:** 2018-12-12

**Authors:** Stefanie Seeling, Franziska Prütz

**Affiliations:** Robert Koch Institute, Berlin, Department of Epidemiology and Health Monitoring

**Keywords:** CHILDREN AND ADOLESCENTS, UTILIZATION OF OUTPATIENT SERVICES, ORTHODONTICS, HEALTH MONITORING, KIGGS

## Abstract

For patients with considerable malfunctions, or where these could potentially develop, statutory health insurers completely cover the cost of correcting malpositionings of the teeth and jaws through orthodontic treatment in Germany. Based on the self-reported information from the second wave of the German Health Interview and Examination Survey for Children and Adolescents (KiGGS Wave 2, 2014-2017), the uptake of orthodontic treatment was analysed. A total of 25.8% of the girls and 21.1% of the boys aged 3 to 17 years are receiving regular orthodontic treatment. Uptake of treatment is very much dependent on age. The highest rates are found among 13-year-old girls (55.0%) and 14-year-old boys (50.8%). Compared to the data from previous KiGGS waves, the trend over approximately ten years has seen a significant increase of orthodontic treatment across all age groups. More health services research and a broader discourse on current treatment practices and their benefits are desirable.

## Introduction

Malpositioned teeth and jaws can be linked to biting, chewing, speaking or breathing difficulties. Orthodontic treatment can correct malpositionings and remediate, alleviate or prevent malfunctions. An early indication for treatment is important to facilitate an appropriate commencement of therapy, and depending on the underlying malpositioning treatment is also only possible within a specific time frame. The dentists offering the treatment, who usually have a specialisation in orthodontics, can choose between different treatment options with removable or fixed braces. It generally takes several years to correct the positioning of teeth and jaws and the final stage of treatment is a stabilisation phase. Certain diagnoses of severe malpositionings may require surgical treatment.

The National Association of Statutory Health Insurance Dentists (KZBV) reports about 7.9 million cases of orthodontic treatment for 2016 which is an increase for the tenth year in a row [1]. As statutory health insurances only cover certain diagnoses and forms of therapy for adults, the overwhelming majority of patients are children and adolescents [2].

In 2003, the Federal Joint Committee (G-BA) established guidelines for orthodontic treatment for the first time, which came into force in 2004 [2]. They determined that statutory health insurance (GKV) would only cover the treatment of existing or imminent malfunctions and not purely cosmetic treatments. As a result, this led to a reduction of expenditure for GKV on orthodontics in the short term but it increased again from 827.7 million Euro in 2006 to around 1,102.9 million Euro in 2016 [1]. GKV allotted around one quarter of the total amount billed to materials and laboratories and three quarters on dentists’ fees [3]. Orthodontic treatment costs in private health insurance totalled 278.1 million Euro [3]. In addition there are out-of-pocket payments of unknown amounts [4].

**KiGGS Wave 2**

Second follow-up to the German Health Interview and Examination Survey for Children and Adolescents

**Data owner:** Robert Koch Institute

**Aim:** Providing reliable information on health status, health-related behaviour, living conditions, protective and risk factors, and health care among children, adolescents and young adults living in Germany, with the possibility of trend and longitudinal analyses

**Study design**: Combined cross-sectional and cohort study

**Cross-sectional study in KiGGS Wave 2**

**Age range:** 0-17 years

**Population:** Children and adolescents with permanent residence in Germany

**Sampling:** Samples from official residency registries - randomly selected children and adolescents from the 167 cities and municipalities covered by the KiGGS baseline study

**Sample size:** 15,023 participants

**KiGGS cohort study in KiGGS Wave 2**

**Age range:** 10-31 years

**Sampling:** Re-invitation of everyone who took part in the KiGGS baseline study and who was willing to participate in a follow-up

**Sample size:** 10,853 participants

**KiGGS survey waves**

► KiGGS baseline study (2003-2006), examination and interview survey
► KiGGS Wave 1 (2009-2012), interview survey
► KiGGS Wave 2 (2014-2017), examination and interview survey

More information is available at www.kiggs-studie.de/english

The medical benefits of orthodontic treatment are currently the subject of much debate. In the comments on its 2017 annual report, Germany’s Supreme Audit Institution (Bundesrechnungshof) declared that both the aim and outcome of treatments have not been sufficiently researched and called for more health services research [4]. This criticism falls into line with the request for more transparency made a couple of years ago by the Advisory Council on the Assessment of Developments in the Health Care System and the German Institute of Medical Documentation and Information [5, 6].

Based on current data from the German Health Interview and Examination Survey for Children and Adolescents (KiGGS Wave 2, 2014-2017), this Fact sheet contributes to the discussion by providing a robust data set on the prevalence (frequency) of uptake of orthodontic treatment. It provides an analysis for 3- to 17-year-old children and adolescents in regular orthodontic treatment. Influencing factors that are taken into account are gender, age and socioeconomic status. In addition with reference to the data from previous KiGGS waves, it is possible to describe trends for 7- to 17-year-old children and adolescents over the course of about ten years.

## Indicator

KiGGS is part of the health monitoring system at the Robert Koch Institute (RKI) and includes repeated, cross-sectional surveys of children and adolescents aged 0 to 17 (KiGGS cross-sectional study) that are representative for Germany. The KiGGS baseline study (2003-2006) was conducted as an examination and interview survey, the first follow-up study (KiGGS Wave 1, 2009-2012) as a telephone-based interview survey and KiGGS Wave 2 (2014-2017) as an examination and interview survey.

A detailed description of the methodology is contained in New data for action. Data collection for KiGGS Wave 2 has been completed in issue S3/2017 as well as KiGGS Wave 2 cross-sectional study – participant acquisition, response rates and representativeness in issue 1/2018 of the Journal of Health Monitoring [7, 8].

KiGGS Wave 2 surveyed the uptake of orthodontic treatment using a written questionnaire. The legal guardians of 3- to 10-year-old children, as well as 11- to 17-year-old children and adolescents themselves were asked ‘Does your child/Do you receive regular orthodontic treatment?’ Respondents could answer either ‘Yes’ or ’No’.

The analyses are based on data from 12,735 children and adolescents (6,425 girls and 6,310 boys) aged 3 to 17 with valid data on uptake of orthodontic treatment. The results are presented as prevalences (frequency) and are stratified according to gender, age and socioeconomic status (SES) [9]. Trends were assessed for 7- to 17-year-old children and adolescents in comparison to the KiGGS baseline study and KiGGS Wave 1 that collected comparable data for the indicator.

The calculations were carried out using a weighting factor that corrects deviations within the sample from the population structure with regard to regional structure (rural area/urban area), age (in years), gender, federal state (as at 31 December 2015), German citizenship (as at 31 December 2014) and the parents’ level of education (Microcensus 2013 [10]). Moreover, the calculation of p-values to demonstrate linear trends across the three KiGGS survey waves using univariate logistic regression was based on age-standardised prevalences (as at 31 December 2015). This article considers prevalences with 95% confidence intervals (95% CI). Prevalences are estimates, the precision of which can be assessed through the use of confidence intervals; wide confidence intervals thereby indicate a greater statistical uncertainty in the results. A statistically significant difference between groups is assumed when the corresponding p-value is smaller than 0.05 with respect to weighting and survey design.

## Results and discussion

KiGGS Wave 2 data shows that 23.4% of 3- to 17-year-old children and adolescents (girls 25.8% and boys 21.1%) are in regular orthodontic treatment (Table 1). From the age of 7 across all age groups a statistically significant higher number of girls receive treatment than boys.

As expected, uptake of orthodontic treatment is very much dependent on age. Reaching 47.6% for girls and 36.1% for boys, prevalence is highest in the 11- to 13-year age group, whereas only very few 3- to 6-year-olds are in orthodontic treatment (Table 1). Uptake increases continuously beyond the age of 3. The highest values are found for 13-year-old girls (55.0%) and 14-year-old boys (50.8%), prevalences then begin to drop (data not shown).

Girls who grow up in families with low SES are in orthodontic treatment significantly less often than girls from families with medium SES. For boys, uptake for the group with low SES is significantly lower than for those with medium or high SES (Table 1).

**Figure 1:**
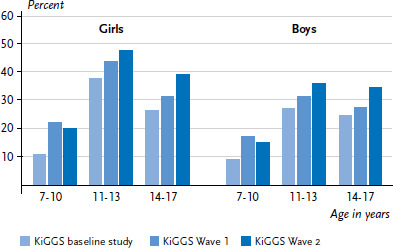
Prevalence of uptake of orthodontic treatment by 7- to 17-year-olds according to gender and age compared to the previous KiGGS Waves (KiGGS baseline study n=5,211 girls, n=5,467 boys; KiGGS Wave 1 n=3,790 girls, n=3,799 boys; KiGGS Wave 2 n=4,797 girls, n=4,598 boys) Source: KiGGS baseline study (2003-2006), KiGGS Wave 1 (2009-2012), KiGGS Wave 2 (2014-2017)

Whereas the prevalence of orthodontic treatment among 7- to 17-year-olds was 22.0% in the KiGGS baseline study, this rose to 28.0% in KiGGS Wave 1 and 31.1% in KiGGS Wave 2 (age-standardised as at 31 December 2015, data not shown). The ten year trend shows a significant increase across all the age groups examined as much for girls as for boys (Figure 1).

KiGGS Wave 2 results show that – depending on age – up to 55.0% of girls and 50.8% of boys in Germany are currently in orthodontic treatment. These results are congruent with KZBV estimates quoted by Germany’s Supreme Audit Institution, according to which over half of all children and adolescents in Germany receive orthodontic treatment [4]. Early orthodontic treatment is already provided to the 3- to 6-year-old age group, even if it is only to a small proportion of children (2.1%).

Using KiGGS Wave 2 data, it is estimated in absolute figures that roughly 2.5 million children and adolescents are in regular orthodontic treatment. That indicates with respect to the nearly eight million treatment cases reported by KZBV for 2016 [1] that not every insured person receives treatment in every quarter of the year, for example due to agreed treatment breaks.

What is noteworthy is the clear differences according to gender. Over the age of seven, girls in Germany of all age groups receive orthodontic treatment more frequently than boys. In other countries, too, girls are in orthodontic treatment more often, even though they are not more affected by malpositioning [11]. This is generally explained by the greater aesthetic demands made by girls and their parents, but also by a greater degree of openness to orthodontic treatment [11-13].

The fact that children and adolescents from families with low SES are less often in orthodontic treatment than their peers from families with medium SES and for boys also with high SES does not reflect actual treatment needs. Adolescents from socially disadvantaged families more frequently have insufficient oral hygiene [14] and less healthy teeth [15] that lead to malpositioning and may also present an indication for orthodontic treatment [5].

The data also shows that the trend for orthodontic treatment has seen a rise over the last ten years for both genders and across all age groups. This is in line with KZBV statistics, according to which the number of treatment cases has increased continuously over this time period [1].

The data analysed on the uptake of orthodontic treatment from the perspective of parents or children and adolescents themselves provides important information on health care provision that goes beyond that provided by claims data. It collects data from people in both statutory and private health insurance and allows connections with socio-demographic and other influencing factors to be made.

Only around 5% of the population has an ideally positioned natural dentition regarding as to the position of teeth in the upper jaw relative to the teeth of the lower jaw [16]. Not all people whose teeth are not ideally aligned, however, require treatment, their teeth are not a medical indication for treatment. In its orthodontic treatment guidelines, the G-BA has determined that the GKV only covers the cost of orthodontic treatment in cases where substantial functional limitations exist or where these could develop. However, the majority of children and adolescents and/or their parents do not seem to perceive functional limitations, they take up treatment based on the recommendation of dentists [17]. Their motivation to undergo a treatment that will take several years to complete is also an expected aesthetic improvement [17, 18].

After the Supreme Audit Institution objected (see above), the German Society of Orthodontists (DGKFO) [19], the Professional Association of German Orthodontists (BDK) [20] and the KZBV [21] all provided written statements. The broad consensus was the need to intensify the health services research regarding orthodontic treatment. For the coming German Oral Health Study (DMS VI), the DGKFO for example plans to add further questions in the field of orthodontics; at the same time the society rejected several criticisms of the Supreme Audit Institution as incorrect [19]. Against the backdrop of an incomplete basis of studies and diverging interests (for example wish-fulfilling medicine regarding aesthetic ideals) an ethically grounded discourse between the actors involved is encouraged [18]. This should also include a discussion of a long-term risk-benefit-assessment of current orthodontic treatment practice.

## Key statements

- Prevalence of uptake of orthodontic treatment is highest in the age group of 11- to 13-year-old girls (47.6%) and boys (36.1%).
- The uptake of orthodontic treatment for girls and boys aged over 7 has increased across all age groups since the KiGGS baseline study (2003-2006).
- On average, more girls undergo orthodontic treatment than boys.
- The uptake of orthodontic treatment for girls and boys with low socioeconomic status (SES) is lower than for their peers with medium SES, for boys also with high SES.

## Figures and Tables

**Table 1:** Prevalence of uptake of orthodontic treatment by 3- to 17-year-olds according to gender, age and socioeconomic status (n=6,425 girls, n=6,310 boys) Source: KiGGS Wave 2 (2014-2017)

%		(95% CI)	%		(95% CI)
**Girls (total)**	25.8	(24.4-27.2)	**Boys (total)**	21.1	(19.7-22.5)
**Age group**			**Age group**		
3-6 Years	2.1	(1.3-3.4)	3-6 Years	2.1	(1.4-3.3)
7-10 Years	20.0	(17.4-22.9)	7-10 Years	15.1	(13.1-17.4)
11-13 Years	47.6	(44.2-50.9)	11-13 Years	36.1	(32.6-39.7)
14-17 Years	39.2	(36.2-42.2)	14-17 Years	34.7	(31.5-38.1)
**Socioeconomic status**			**Socioeconomic status**		
Low	22.1	(18.8-25.9)	Low	15.9	(12.3-20.2)
Medium	26.4	(24.6-28.4)	Medium	22.2	(20.5-23.9)
High	26.3	(24.0-28.8)	High	22.6	(19.9-25.5)
**Total (girls and boys)**	23.4	(22.4-24.4)	**Total (girls and boys)**	23.4	(22.4-24.4)

CI=Confidence interval
